# Treatment of mice with a ligand binding blocking anti-CD28 monoclonal antibody improves healing after myocardial infarction

**DOI:** 10.1371/journal.pone.0227734

**Published:** 2020-04-16

**Authors:** Nadine Gladow, Claudia Hollmann, Gustavo Ramos, Stefan Frantz, Thomas Kerkau, Niklas Beyersdorf, Ulrich Hofmann

**Affiliations:** 1 Comprehensive Heart Failure Center, University Hospital Würzburg, Würzburg, Germany; 2 Department of Internal Medicine I, University Hospital Würzburg, Würzburg, Germany; 3 Institute for Virology and Immunobiology, University of Würzburg, Würzburg, Germany; Albert Einstein College of Medicine, UNITED STATES

## Abstract

Both conventional and regulatory CD4^+^ T-cells rely on costimulatory signals mediated by cell surface receptors including CD28 for full activation. We showed previously that stimulation of CD4^+^ Foxp3^+^ regulatory T-cells by superagonistic anti-CD28 monoclonal antibodies (mAb) improves myocardial healing after experimental myocardial infarction (MI). However, the effect of ligand binding blocking anti-CD28 monoclonal antibodies has not yet been tested in this context. We hypothesize that ligand blocking anti-CD28 mAb treatment might favorably impact on healing after MI by limiting the activation of conventional CD4^+^ T-cells. Therefore, we studied the therapeutic effect of the recently characterized mAb E18 which blocks ligand binding to CD28 in a mouse permanent coronary ligation model. E18 or an irrelevant control mAb was applied once on day two after myocardial infarction to wildtype mice. Echocardiography was performed on day 7 after MI. E18 treatment improved the survival and reduced the incidence of left ventricular ruptures after experimental myocardial infarction. Accordingly, although we found no difference in infarct size, there was significantly less left ventricular dilation after E18 treatment in surviving animals as determined by echocardiography at day 7 after MI. In sham operated control mice neither antibody had an impact on body weight, survival, and echocardiographic parameters. Mechanistically, compared to control immunoglobulin, E18 treatment reduced the number of CD4^+^ T-cells and monocytes/macrophages within the infarct and periinfarct zone on day 5. This was accompanied by an upregulation of arginase which is a marker for alternatively differentiated macrophages. The data indicate that CD28-dependent costimulation of CD4^+^ T-cells impairs myocardial healing and anti-CD28 antibody treatment constitutes a potentially clinically translatable approach to improve the outcome early after MI.

## Introduction

Foxp3^+^ regulatory T-cells (Tregs) infiltrating the infarcted myocardium have been shown to favorably regulate macrophage differentiation, monocyte recruitment, extracellular matrix *de novo* formation, and angiogenesis [1–3]. These processes play central roles in myocardial healing after myocardial infarction (MI). On the other hand, in murine models of non-ischemic, pressure-overload induced heart failure, activated conventional CD4^+^ T-cells infiltrate the heart and are crucial promoting factors for dysfunctional cardiac fibrosis, hypertrophy, and remodeling [4]. Furthermore, adverse effects of conventional Foxp3^-^ CD4^+^ T-cells on chronic remodeling of the heart after experimental MI have been demonstrated recently [5]. However, whether and how conventional CD4^+^ T-cells affect the pathophysiology of early myocardial healing after MI has not yet been explored.

CD28 is a homodimeric cell surface receptor that acts as the main co-stimulator of primary T-cell responses. It is expressed on virtually all T-cells in rodents including CD4^+^ T-cells. For complete activation, besides binding to their cognate antigen presented on major histocompatibility complex molecules on antigen-presenting cells by their T-cell receptor, T-cell require costimulatory signals, e.g. provided by the engagement of CD28 through binding of CD80/86. In the following, T cells start to proliferate and, depending on the local cytokine milieu, differentiate into various types of effector cells. As the costimulatory molecule CD28 is a key modulator of T-cell activation, it constitutes an attractive therapeutic target in T-cell dependent diseases [6]. To this end, monoclonal antibodies have been developed which either block ligand binding or superagonistically stimulate CD28 (reviewed in [6]). Application of monoclonal antibodies (mAb) with specificity for CD28 blocking B7 ligand binding to CD28 into healthy mice leads to a decline in CD4^+^ Foxp3^+^ regulatory T cells (Tregs) [7] as these depend on constant CD28 stimulation for their maintenance [8–11]. During an immune response, CD4^+^ Foxp3^-^ conventional T cells (Tconvs), however, heavily depend on CD28 costimulation for clonal expansion, while Tregs are largely independent of CD28 costimulation in the inflammatory milieu of an evolving immune response. Therefore, blocking CD28 costimulation with E18 e.g. during a superantigen-driven T cell response or in acute Graft versus Host Disease more strongly inhibits the expansion of Tconvs than that of Tregs leading to an increase in Treg frequencies among CD4^+^ T cells [7]. MAb E18 was thus capable of directly suppressing Tconv activation by blocking costimulation of these cells and, indirectly, by increasing frequencies of Tregs among CD4^+^ T cells. Upon superagonistic monoclonal anti-CD28 injection Tregs are preferentially activated over Tconvs and expanded also leading to potent inhibition of (pathogenic) Tconv cell responses [12–17].

Complementing our previous results on the central role of Tregs in myocardial healing [18] in the present work we focused on the therapeutic manipulation of Foxp3^-^ CD4^+^ conventional T-cells. We show that the anti-CD28 monoclonal antibody E18 blocking B7 ligand binding to CD28 reduces the infiltration of the infarcted myocardium by CD4^+^ T-cells and improves the early healing processes. Our results point to a significant role of heart infiltrating conventional CD4^+^ T-cells after myocardial infarction.

## Materials and methods

### *In vivo* mouse experiments

Male C57B6/J mice (age 8–12 weeks, body weight 26-30g) were supplied by Envigo and housed under specific pathogen free conditions and under controlled environmental conditions. The animals had access to standard diet and water ad libitum. All mice were allowed to acclimatize to their environment for at least one week.

Myocardial infarction was induced by permanent ligation of the left anterior descending coronary artery. For this, the mice (78 in total] were anesthetized using 3–4% (vol./vol.) isoflurane, intubated, and ventilated with oxygen/ 0.8–1.2% isoflurane. During sham surgery (19 mice), thoracotomy was conducted without ligating the vessel. For analgesia, all mice undergoing surgeries were injected with buprenorphine (0.1 mg/kg s.c.; Bayer, Cat# 796–298) before surgery and at least twice a day during the following days. Mice were scored twice according to a multi-dimensional scoring system for assessment of pain and animal condition. The scoring included the condition of fur, respiration, muscle tonus, behavior, and the condition of the surgical wound. For each dimension the scoring gives 0 to 20 points. The overall score differentiates no (0 points), low (1–9 points), intermediate (10–19 points), and high burden (>19 points). In the latter case immediate euthanasia is mandatory but no mice met the criteria for humane endpoints. Within the group of MI operated animals 10 died within the first 16 hours due to the operation itself, another 8 animals died from sudden death during the following days without reaching a human endpoint criterium. Sudden death due to arrhythmias and left ventricular rupture without any auguries is a typical finding in the MI model.

Mice were randomly assigned to the treatment groups, either anti-CD28 monoclonal antibody clone E18 (250 μg Exbio, Cat#10-CM036-BUCK) or isotype-control IgG2b antibody with irrelevant specificity, clone MPC-11, (250 μg BioXCell, Cat# BE0086) and were injected intraperitoneally on day two after MI. Antibodies were validated for specificity in a previous work [7]. Animals dying within 16 hours after surgery and with an infarct size below 20% were excluded post hoc from analysis. Animals were sacrificed with carbon dioxide for cellular and molecular analysis on day 5 or day 7 after MI.

Echocardiography was performed by a blinded technician in spontaneously respirating animals under light isoflurane (about 1%) anesthesia.

Approval was granted for all animal procedures by the local institutional ethics review board (Regierung von Unterfranken). All animal procedures were performed conforming to the guidelines from Directive 2010/63/EU of the European Union on the protection of animals used for scientific purposes.

### Quantitative real-time PCR

Messenger RNA (mRNA) was extracted from the spleen of non-reperfused mice using the RNeasy Mini Kit (Qiagen, Cat# 74104) according to the manufacturer’s instructions. One microgram of mRNA was transcribed to complimentary DNA (cDNA) with the iScript kit (BioRad, Cat# 1708891). TaqMan primers (Life Technologies, Tnf, Mm00443260_g1; Nos2, Mm00440488_m1; IL-1b, Mm0043228_m1; IFNγ, Mm01168134_m1; Arg, Mm00475988_m1; Col1a1, Mm00483888_m1; Col3a1, Mm01254476_m1; Spp1, Mm00436767_m1; IL-10, Mm00439615_g1; TGFβ1, Mm01178820_m1; Timp2, Mm00441825_m1; Mmp9, Mm00442991_m1; Mmp2, Mm00439498_m1) were employed for the quantitative real-time PCR (Applied Biosystems). Results are expressed as Ct values normalized to the housekeeping gene *Gapdh*. For real-time PCR analysis of FACS-sorted cells, the RNeasy Micro Kit (Qiagen, Cat# 74004) was applied and the mRNA was amplified using the REPLI-g WTA Single Cell kit (Qiagen, Cat# 150065) according to the manufacturer´s protocol.

### Fluorescence activated cell sorting analysis

To analyze leukocyte populations after MI mediastinal lymph nodes, hearts and spleens were isolated and single cell suspensions were prepared. Splenocytes were depleted of erythrocytes by incubating with a lysis buffer (Biolegend, Cat# 420301) for 10 minutes at RT. For FACS staining of heart cells, heart scar tissue was dissected into small pieces and digested with collagenase (1000 U/ml; Worthington, Cat# 41H12763) at 37°C for 25 minutes. Stainings were performed with up to 10^6^ trypan-blue negative cells in 25μl of PBS/0.1% BSA/0.02% NaN3. Fcγ II/III receptors were pre-blocked by incubation with saturating amounts of cell culture supernatant of the clone 2.4G2 (anti-Fcγ receptor II/III monoclonal antibody). For cell surface labelling antibodies were added to the blocking solution (25μl) and incubated for 15 min at 4°C. For intracellular staining, cell surface labelled cells were fixed for 30 min at room temperature with fixation buffer (eBioscience, Cat# 88-8824-00) and stained with intracellular antibodies in permeabilization buffer (eBioscience, Cat# 00-8333-56) for 45 min at room temperature. For analysis of intracellular cytokines, cells were stimulated ex vivo with phorbol-12-myristate-13-acetate (PMA, Sigma, Cat# 19–144) and ionomycin (Sigma, Cat# I9657) in the presence of monensin (BioLegend, Cat# 420701) for 4 hours at 37°C followed by intracellular stainings of cells as described above. To check for the viability of T-cells, cells were stained with Annexin V (Biolegend Cat# 640905) and viability dye eFluor 780 (eBioscience, Cat# 65-0865-14). To define T-cell subsets the following antibodies were used: CD45 Brilliant Violet 510 (BioLegend, Cat# 103137), CD3 Pac Blue (eBioscience, Cat# 48003182), CD3 PE Cy7 (BioLeged, Cat# 102016), CD4 APC-Cy7 (BioLegend Cat#100525),CD3 APC CD25 PE-eFluor 610 (clone PC61.5, Invitrogen, Cat# 61-0251-80), CD44 FITC (BioLegend, Cat# 103021), CD62L PE (BioLegend 104407), Foxp3 PerCp5.5 (eBioscience, Cat# 45-5773-80),Ki67 eFluor 660 (eBioscience, Cat# 50-5698-80). To define myeloid subsets we used: CD45 FITC (BD Biosciences, Cat# 553080),CD11b Alexa Fluor 488 (BioLegend, Cat# 101207), CD11b APC Cy7Ly6C PE-Cy7 (BioLegend, Cat# 128017).

To calculate absolute cell numbers counting beads (Invitrogen, Cat# C36950) were included. Cells were measured on an LSR II flow cytometer (BD Biosciences) and analyzed with the FlowJo Software (TreeStarInc, V10.4.1, https://www.flowjo.com/). Cell Sorting was performed using a FACS Aria III (BD Biosciences).

### Histology

Sections of mouse myocardium were fixed in 4% paraformaldehyde overnight and embedded in paraffin. For determination of infarct size by manual planimetry, collagen was stained by picrosirius red on 7-μm sections. Extracellular matrix staining (Ladewig) was performed on day 5 after MI on 7-μm sections using the Ladewig kit from Morphisto. Alpha smooth muscle actin (α-SMA) stainings were conducted on 7-μm sections using the Dako ARK^™^ Peroxidase Kit in combination with anti-α-SMA monoclonal antibody clone 1A4 (Dako, Carpinteria) and, subsequently, stained with hematoxylin. Semi-quantitative grading for expression was performed independently by two researchers blinded for genotype on 400-fold magnified light microscopic fields. Grading was defined as follows: O indicates absence of positively stained cells on all fields examined, whereas 4 indicates a high density of positive cells in each field. 1 indicates positive cells in at least one field, 2 and 3 means low and high density of positive cells, respectively, on more than one microscopic field. For light microscopy, slides were dehydrated and mounted. Pictures were acquired on a Zeiss Axioskiop 2 plus microscope using Spot Software 5.0 (Diagnostics Instruments, Inc, Sterling Heights).

### Statistical analyses

The results are shown as mean values ± standard errors of the mean standard errors (SD) or standard errors of the mean (SEM) if individual data points represent technical replicates. Statistical calculations were done based on a two-tailed t-test or, in case of multiple comparisons, with one-way ANOVA with post hoc Sidak tests if not stated otherwise in the figure legends (tests performed with Graph Pad Prism software, V6, https://www.graphpad.com/scientific-software/prism/). Survival analysis was performed by a Logrank Test (Mantel-Cox). Data were considered to be significant with p < 0.05. The data and statistical analysis comply with the recommendations on experimental design and analysis in pharmacology [19].

## Results

### Ligand blocking anti-CD28 monoclonal antibody treatment prevents left ventricular dilation and rupture after MI

**Fig 1:**
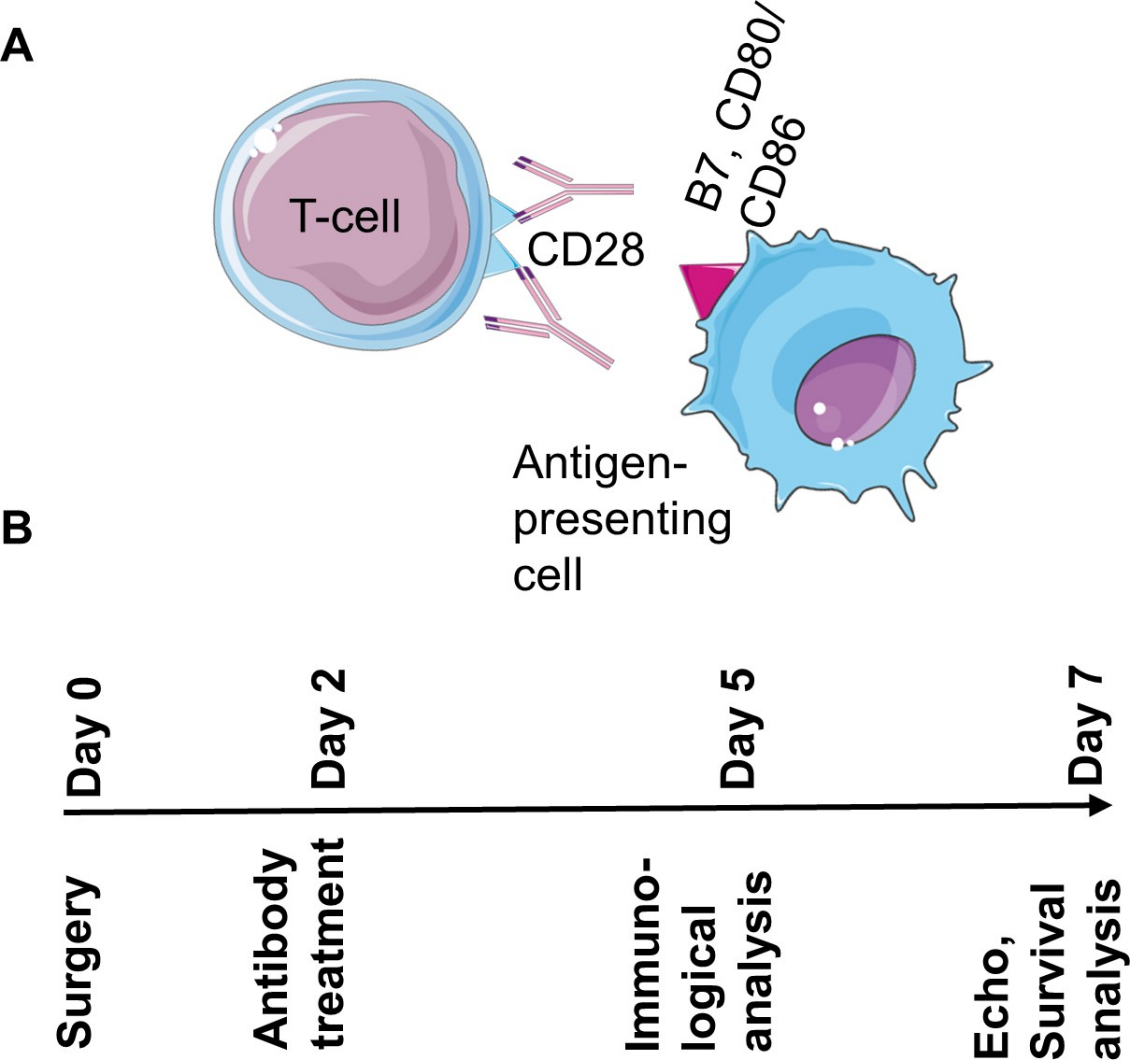
The anti-CD28 antibody E18 blocks the interaction of costimulatory B7 molecules on antigen-presenting cells with the CD28 receptor on T-cells (A). Study protocol (B). At day 2 after surgery animals were randomized to IgG or anti-CD28 treatment and followed until day 5 or 7 after surgery for immunological and gene expression analysis or echo, respectively.

**Fig 2:**
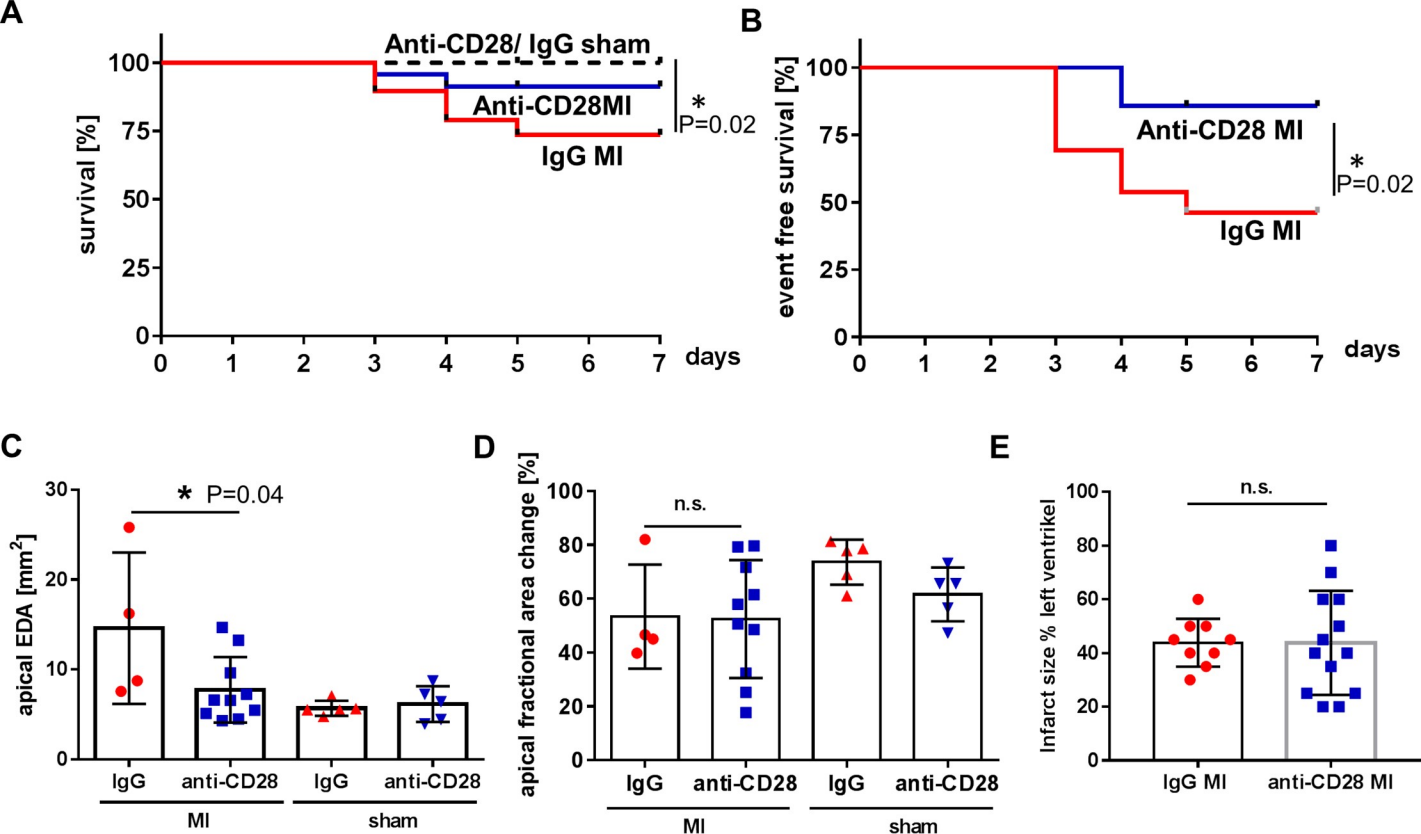
Treatment with anti-CD28 mAb E18 significantly enhanced survival compared to IgG control treatment (C: n = 19 IgG MI, n = 23 anti-CD28 MI, n = 11 IgG sham, n = 10 anti-CD28 sham, P<0.05, Logrank Test, A). The rupture rate within the 7 days study was significantly lower in anti-CD28 treated animals (n = 19 IgG MI, n = 23 anti-CD28 MI, P<0.05, Logrank Test, B). The end-diastolic area was significantly smaller in anti-CD28 treated animals (n = 4 IgG MI, n = 10 anti-CD28 MI, n = 5 IgG sham, n = 5 anti-CD28 sham, *p<0.05, ANOVA, means±SEM, C), whereas fractional area change (n = 4 IgG MI, n = 10 anti-CD28 MI, n = 5 IgG sham, n = 5 anti-CD28 sham, means±SEM, D) and infarct size (determined by histology on day 5) (n = 9 IgG MI, n = 13 anti-CD28 MI, means±SEM, E) were not different.

Animals were randomly allocated to treatment with a single dose of the ligand binding inhibiting anti-CD28 mAb E18 or a mAb of the same isotpye (IgG2b), but irrelevant specificity, on day two after myocardial infarction (MI). The anti-CD28 antibody [7] prevents costimulatory ligands expressed on antigen-presenting cells from binding to the T-cell surface molecule CD28 (Fig 1). Mice treated with mAb E18 showed a better survival compared to control IgG treated animals. Fig 2A shows the survival curves of all animals, including animals that were euthanized at day 5 and 7 for pre-specified analyses. Left ventricular rupture is a major cause of death occurring typically between days 3 and 7. Ventricular ruptures were identified by necropsy performed in all animals that had died spontaneously.

In the group of animals that were assigned to the analysis of clinical endpoints on day 7 after MI there were significantly less left ventricular rupture events in anti-CD28 treated animals than in control IgG treated ones (Fig 2B). In accordance to these findings surviving animals showed significantly less left ventricular dilation (Fig 2C) whereas there was no difference in left ventricular systolic function on day 7 (Fig 2D, S1 Table). The infarct sizes, determined on microscopic sections of the left ventricle (Fig 2E), were comparable in both treatment groups. In sham operated animals there was no difference between treatment groups (S1 Table). Collectively, mAb E18 treatment carried out at day two after MI prevented left ventricular ruptures and dilation.

### Anti-CD28 treatment reduces the number of monocytes/macrophages and CD4^+^ T-cells in the infarct zone

**Fig 3:**
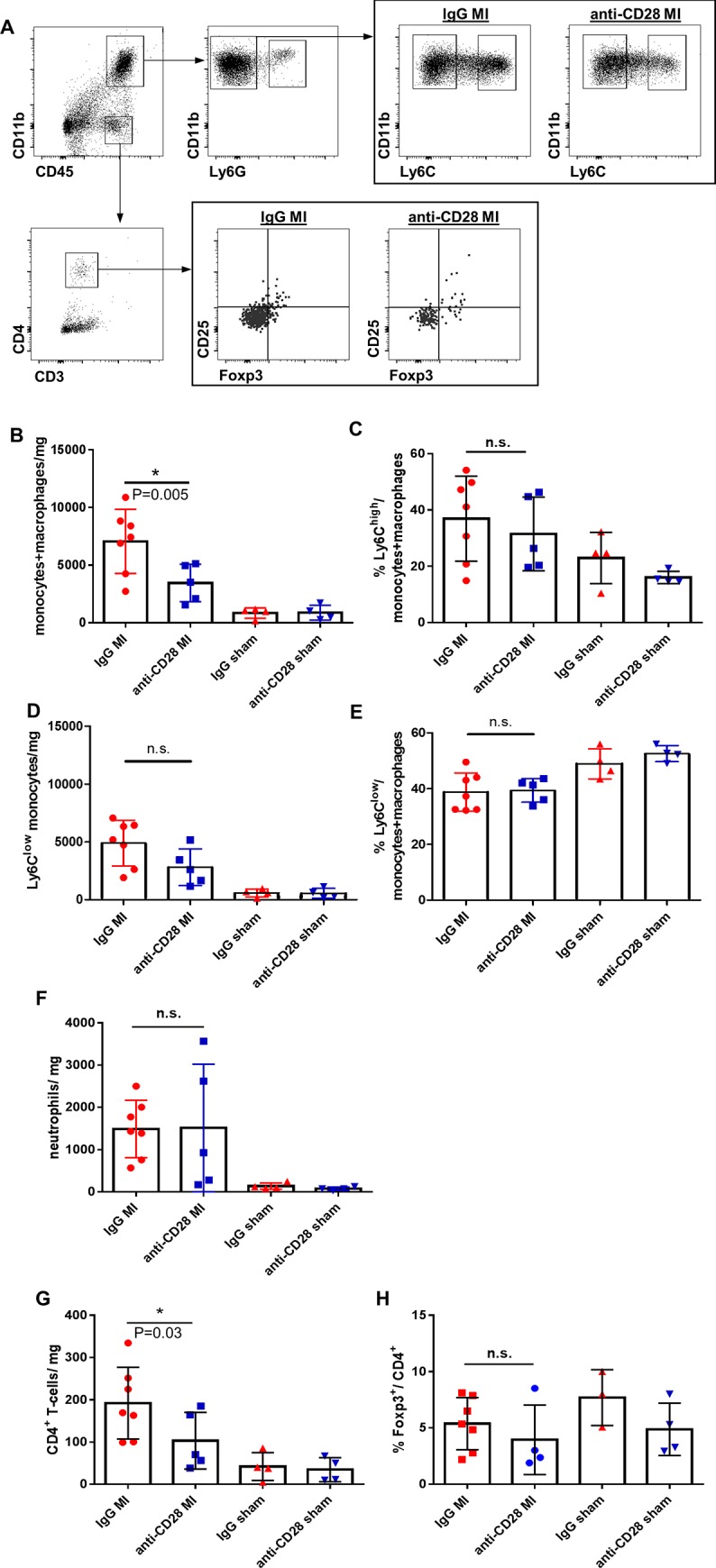
Flow cytometric gating strategy for the analysis of myocardial leukocyte subpopulations (A). Quantitative analysis of myeloid cells (B-F) and CD4^+^ T-cells within the infarct zone (G, H) on day 5 after MI (* P<0.05 ANOVA, means±SD). B-G: n = 7 IgG MI, n = 5 anti-CD28 MI, n = 4 IgG sham, n = 4 anti-CD28 sham; H: n = 7 IgG MI, n = 4 anti-CD28 MI, n = 3 IgG sham, n = 4 anti-CD28 sham.

Around day 5 after MI relevant numbers of T-cells appear and in parallel macrophages undergo a switch towards a pro-healing phenotype within the infarct zone. Therefore, we analyzed the leukocytes within the infarct and peri-infarct zone by flow cytometric analysis (Fig 3A) on day 5. Increased numbers of CD11b^+^ myeloid cells, including monocytes, macrophages, and neutrophils were found in MI vs sham operated animals. Upon treatment with anti-CD28 or control IgG antibody there were significantly less monocytes/ macrophages in the infarct zone of anti-CD28 treated mice (Fig 3B). However, the anti-CD28 treatment did neither change the frequency of proinflammatory Ly6C^high^ monocytes and Ly6C^low^ monocytes/macrophages within the monocyte/macrophage compartment nor the absolute cell numbers of neutrophils (Fig 3C–3F). The total number of CD4^+^ T-cells was significantly lower in the anti-CD28 group (Fig 3G) compared to the control IgG group after MI. Moreover, the frequency of Foxp3^+^ cells within the CD4^+^ T-cell subset was not different between the treatment groups after MI (Fig 3H) implicating that both regulatory and conventional T-cells were reduced. In sham operated animals anti-CD28 treatment neither caused changes in T-cell numbers nor in cell frequencies within the myocardium.

### Effect of anti-CD28 treatment on T-cells in heart draining lymph nodes

**Fig 4:**
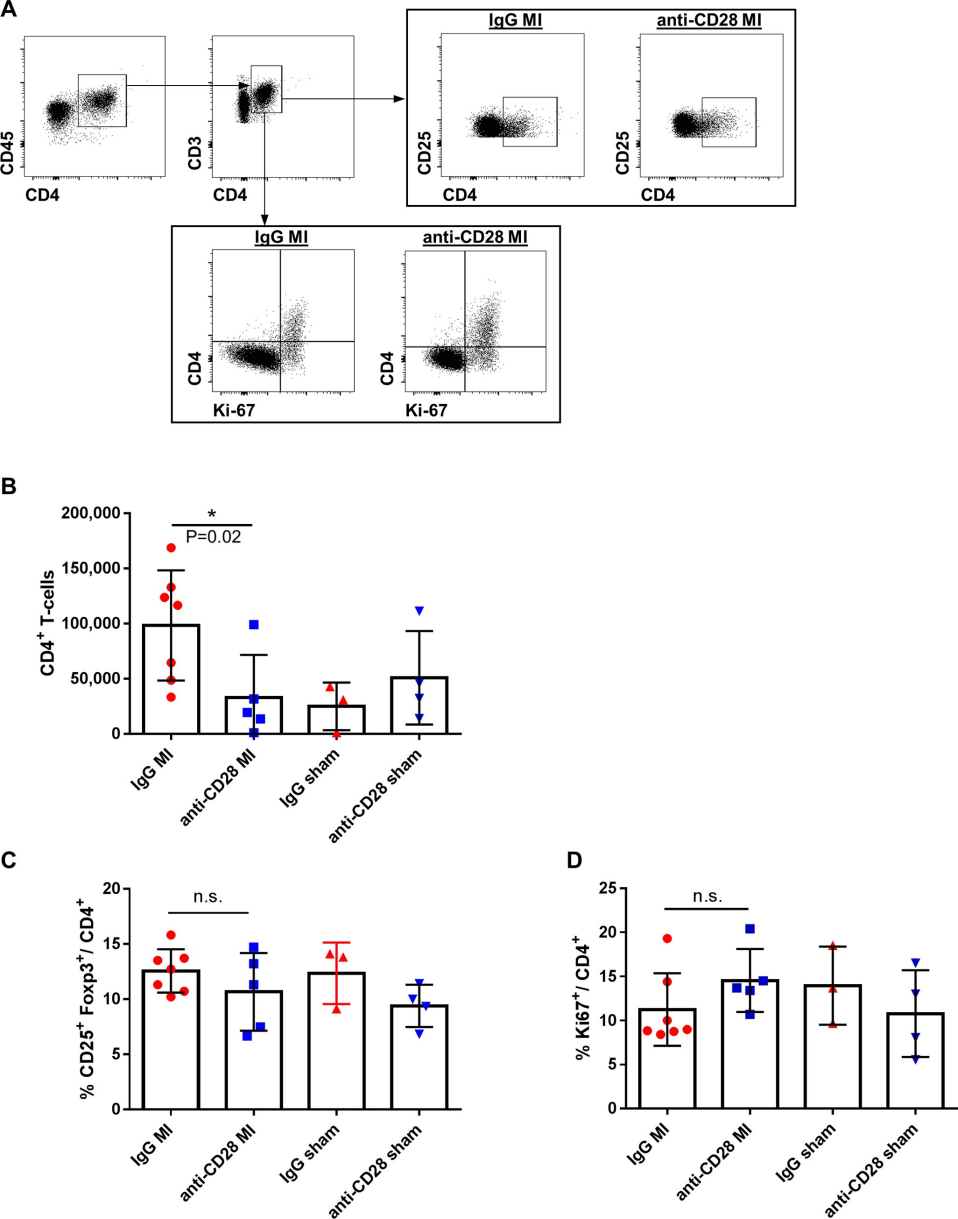
Flow cytometric gating strategy (A) and quantitative analysis of CD4^+^ T-cell subsets in mediastinal lymph nodes (B-D) after MI or sham operation and anti-CD28 or IgG mAb administration 5 days after surgery. (* P<0.05 ANOVA, means±SD). B-D: n = 7 IgG MI, n = 5 anti-CD28 MI, n = 3 IgG sham, n = 4 anti-CD28 sham; * P<0.05 ANOVA, means±SD.

To further dissect whether T-cell activation in secondary lymphoid organs or recruitment into the infarcted heart were responsible for the reduced numbers of CD4^+^ T-cells in the heart after MI we studied the impact of the anti-CD28 antibody treatment on the CD4^+^ T-cell compartment in heart draining lymph nodes as the T-cell activation process in response to MI mainly takes place there [20]. On day 5, the absolute number of CD4^+^ T-cells was significantly lower in anti-CD28 treated animals after MI, whereas there was no treatment effect in sham operated animals (Fig 4A and 4B). The reduction in CD4^+^ T-cells in heart draining lymph nodes was neither associated with differences in the frequency of Foxp3^+^ CD4^+^ T-cells (Fig 4C) nor with a decrease in the number of proliferating (Ki67^+^) CD4^+^ T-cells (Fig 4D). Further, the treatment did not influence the effector/memory subset distribution determined by the surface markers CD62L and CD44 (S1 Fig) in mediastinal lymph nodes.

### Treatment effect on inflammatory and extracellular matrix gene expression

**Fig 5:**
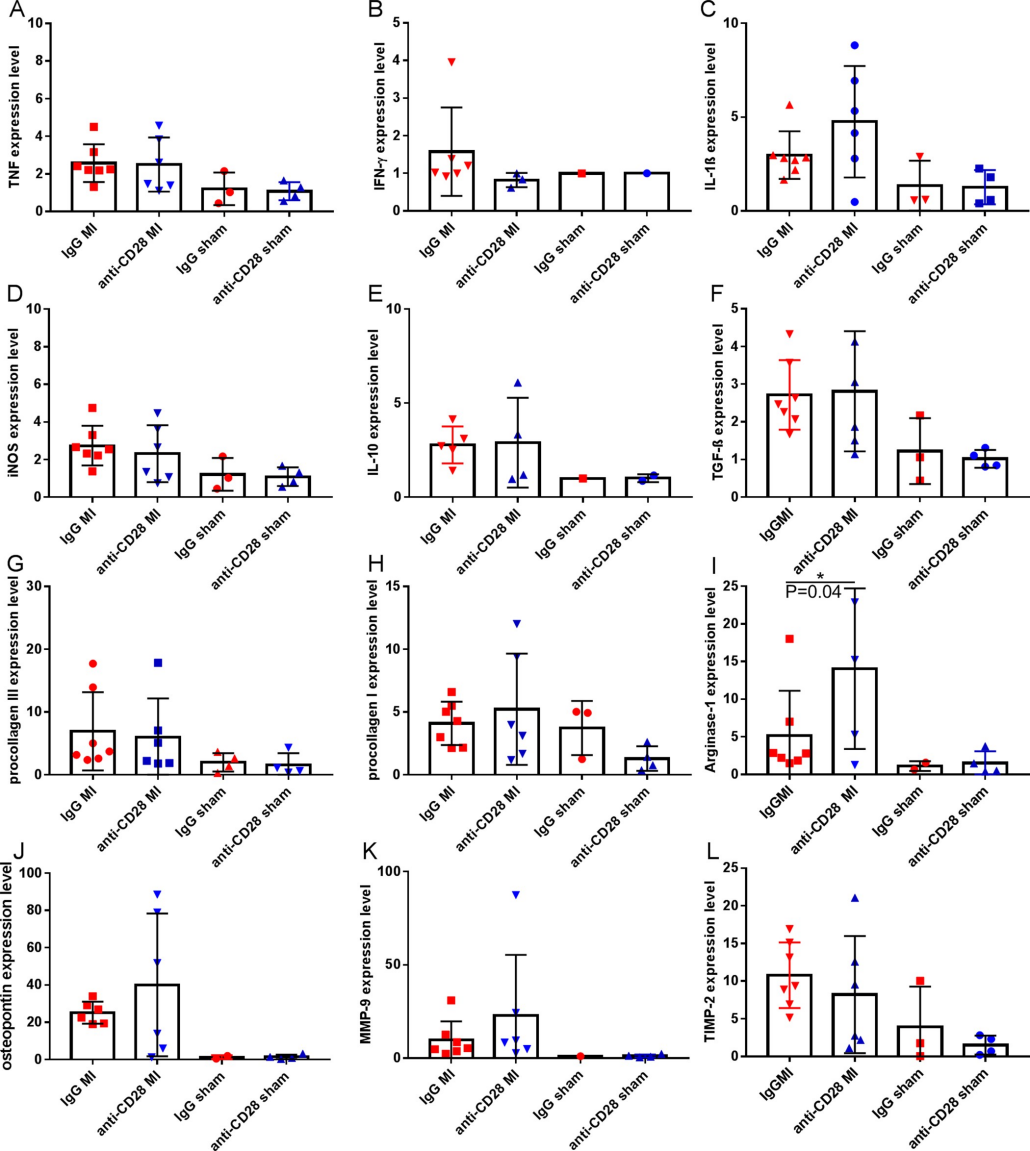
Quantitative real-time PCR analysis reflecting the relative transcription of proteins representing inflammation (A-D), pro-fibrotic/ anti-inflammatory proteins (E-J), and matrix-protease activity (K, L) at 5 days after MI or sham surgery. Transcripts reflecting arginase 1 expression were significantly upregulated in the anti-CD28 vs IgG treatment group (*P<0.05 ANOVA, followed by Dunn’s multiple comparison test comparing the MI groups, means±SD). A-L: n = 5–7 IgG MI, n = 3–6 anti-CD28 MI, n = 1–3 IgG sham, n = 1–4 anti-CD28 sham.

The improved preservation of the left ventricular geometry and the reduced incidence of left ventricular rupture events observed in the anti-CD28 group point to an effect on the extracellular matrix turnover during the early healing phase. In the infarct boarder zone of IgG treated animals there were more leukocytes and there seemed to be less and disarrayed collagen fibers (representative Ladewig staining, S2A Fig). However, no significant difference in myofibroblast activation as analyzed by a α-SMA immunohistological staining (S2B and S2C Fig) became evident 5 days after MI surgery in anti-CD28 vs IgG treatment groups. In addition, we performed quantitative PCR analysis of transcripts related to extracellular matrix remodeling as well as macrophage and myofibroblast differentiation (Fig 5A). Arginase, Osteopontin, and MMP-9 showed a more than 1.5-fold upregulation and Interferon-γ a respective downregulation when comparing the anti-CD28 and IgG treatment groups after MI. However, none of those genes were differentially expressed in the respective sham operated treatment groups. After statistical analysis accounting for multiple testing of the MI animals comparing the anti-CD28 with the control IgG treatment groups, only arginase expression (Fig 5I) turned out to be significantly upregulated in the infarct zone of anti-CD28 mAB treated animals. As arginase and osteopontin are upregulated in alternatively differentiated macrophages during the healing phase [18] this expression pattern indicates that CD28 costimulation on T-cells impacts on the macrophage number and differentiation.

## Discussion

Our data presented here show that blockade of T-cell co-stimulation by mAb E18 administered two days after MI prevented left ventricular dilation and ruptures early after the ischemic event. This led to an overall enhanced survival of mice treated with mAb E18 after MI.

We were the first to show that activated CD4^+^ T-cells improve myocardial healing [20]. In subsequent studies we and others could pinpoint the salutary effects of CD4^+^ T-cells during the healing phase after MI to the Foxp3^+^ T-regulatory cell subset [18, 21]. These cells interact with monocytes and macrophages in the myocardium, attenuate monocyte recruitment, and promote their pro-healing differentiation. Recently, we showed that stimulation of T-regulatory cell activation on day two after MI protects against left ventricular rupture and left ventricular dilation [18]. However, conventional CD4^+^ T-cells are by far more prevalent than Foxp3^+^ T-cells in the infarcted myocardium. They have been described to promote myocardial remodeling [5] but their therapeutic targeting during the early phase after MI has not yet been studied. Therefore, we now focused on therapeutic interference with the activation process of conventional T-cells early after MI.

A hint that costimulatory CD28 signaling might be critical during myocardial healing was provided by a recent publication reporting that CD28 deficient mice show impaired healing due to defective extracellular matrix formation after experimental MI [22]. However, this study did not clarify, if the observed effects were due to disturbed activation of conventional T-cells or, more likely, substantially reduced numbers of natural Tregs in CD28 deficient mice.

In the present study, we found that application of the ligand blocking anti-CD28 mAb E18 reduced the numbers of CD4^+^ T-cells within the infarcted myocardium while the proportion of conventional and regulatory T-cells was unchanged in both, the infarcted myocardium and in the draining lymph nodes. In parallel, the number of CD4^+^ T-cells in heart-draining lymph nodes after MI but not after sham surgery or in spleen was reduced in anti-CD28 mAb treated animals after MI. This most likely points to decreased activation of T-cells and subsequently less recruitment to the infarct zone in anti-CD28 mAb treated animals, although we could not find differences in the frequency of proliferating CD4^+^ T-cells between the treatment groups. However, we cannot further exclude that anti-CD28 treatment interfered with CD28-induced migration of T-cells [23] which might have contributed to less accumulation of CD4^+^ T-cells in the infarcted myocardium.

Monocyte derived macrophages by far outnumber T-cells in the infarcted myocardium. Anti-CD28 treatment was associated with less monocytes/macrophages in the infarct zone. Moreover, we detected significantly increased expression of arginase in the infarct zone of anti-CD28 treated animals. Arginase activity promotes collagen synthesis by converting L-arginine into L-ornithine, an intermediate metabolite in the pro-collagen synthesis and thereby stabilizes the myocardial scar zone. Arginase is expressed in alternatively activated macrophages [24]. We have previously shown that activated Foxp3^+^ CD4^+^ T-cells promote myocardial healing by inducing a differentiation state in myocardial macrophages that is characterized by upregulation of proteins including arginase and osteopontin. Our data indicate that conventional CD4^+^ T-cells activated by a CD28 mediated costimulation pathway interfere with this alternative macrophage activation. Hence, attenuated T-cell and macrophage driven local inflammation might most likely both have contributed to the beneficial effect of the anti-CD28 mAb treatment approach.

## Conclusions

For modulation of antigen specific T-cell responses the surface molecule CD28 constitutes an attractive target molecule. Antibodies against this molecule that are capable of either blocking [25, 26] conventional T-cell activation, as tested here, or superagonistic anti-CD28 mAbs which are boosting the Foxp3^+^ T-cell compartment, have been tested in early clinical studies and proved to be save [27]. The superagonistic mAb approach has been shown to be beneficial in experimental myocardial infarction models before [18, 28]. This study is the first to show that inhibiting CD28 costimulation with an anti-CD28 mAb blocking binding of B7 ligands positively influences healing and early survival of mice after MI. Blockade of costimulatory molecules is a clinically feasible treatment approach that might help to improve healing and prevent from subsequent dysfunctional left ventricular remodeling after MI. Therefore, the approach deserves validation in preclinical studies with a longer follow up. Still one cannot decide which of the aforementioned approaches targeting CD28 is more promising. Data from clinical studies are required to answer this finally.

## Supporting information

S1 Table Echocardiographic analysis of anti-CD28 or control IgG treated mice on day 7 after myocardial infarction (MI) or sham surgery (n = 5 IgG MI, n = 10 anti-CD28 MI, n = 5 IgG sham, n = 5 anti-CD28 sham; means±SEM). End-systolic area (ESA, mm^2^), end-diastolic area (EDA, mm^2^), end-systolic diameter (ESD, mm), end-diastolic diameter (EDD, mm), fractional area change (FAC, %).
(PPTX)

S1 Fig CD4^+^ T-cell subsets in mediastinal lymph nodes on day 5 after MI. Representative plot and quantitative analysis of the four quadrants (B-E: n = 4 IgG MI, n = 5 anti-CD28 MI; all P> 0.05 between treatment groups, means±SD).
(PPTX)

S2 Fig Histological characterization of the infarct zone of anti-CD28 or IgG treated mice 5 days after MI surgery. Representative Ladewig stainings of cardiac sections show collagen fibers in the infarct boarder zone in blue (red arrows in A). B: Myofibroblasts were immuno-stained with α-smooth muscle actin (brown). C: The semi-quantitative scoring of α-SMA showed no significant difference between anti-CD28 and IgG treated mice (n = 10 IgG MI, n = 11 anti-CD28 MI; n.s., t-test, means±SD).
(PPTX)
