# Correction: Treatment of mice with a ligand binding blocking anti-CD28 monoclonal antibody improves healing after myocardial infarction

**DOI:** 10.1371/journal.pone.0235947

**Published:** 2020-07-06

**Authors:** Nadine Gladow, Claudia Hollmann, Gustavo Ramos, Stefan Frantz, Thomas Kerkau, Niklas Beyersdorf, Ulrich Hofmann

There is an error in the labels in Fig 4A. The authors have provided the corrected version here.

**Fig 4:**
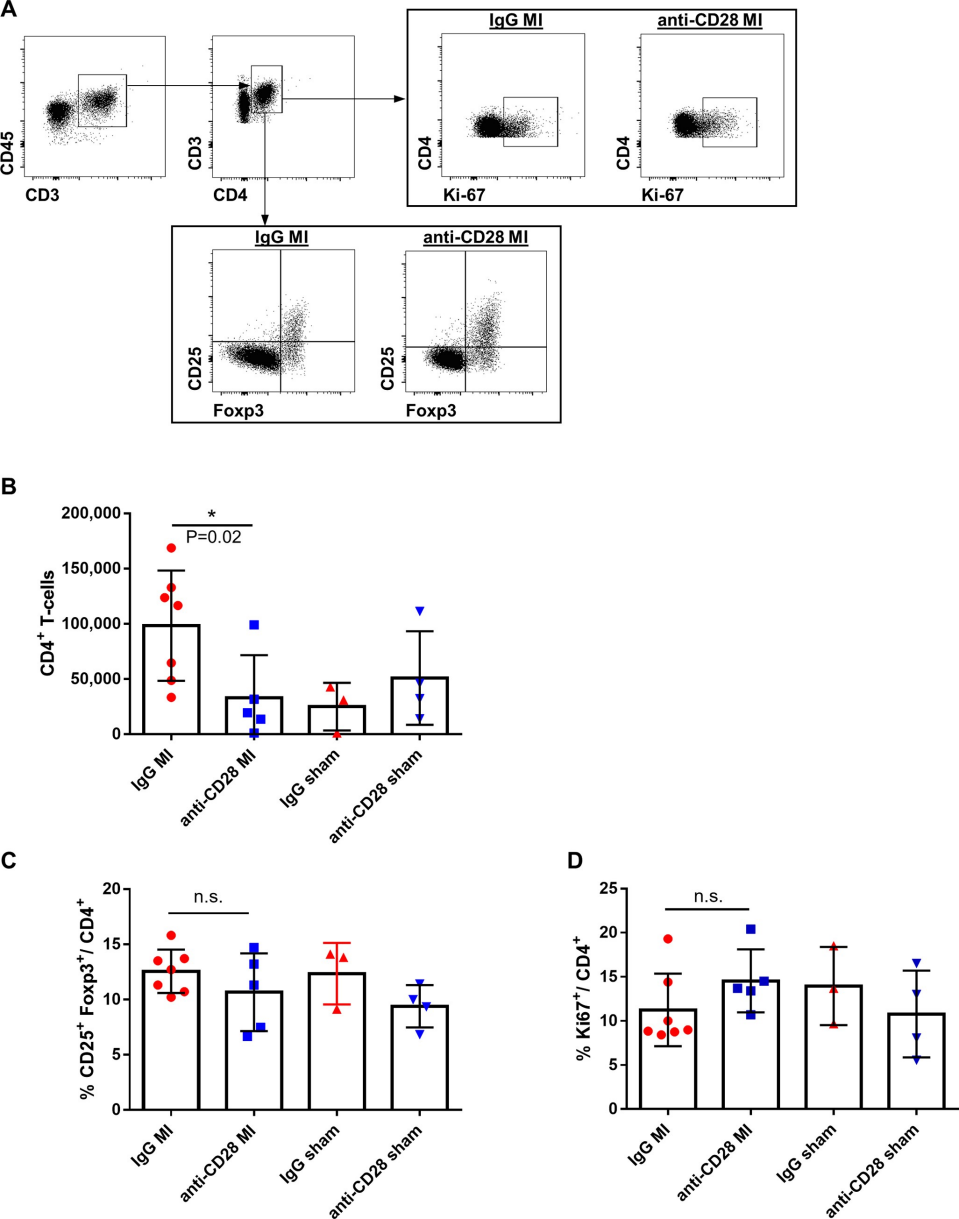
Flow cytometric gating strategy (A) and quantitative analysis of CD4+ T-cell subsets in mediastinal lymph nodes (B-D) after MI or sham operation and anti-CD28 or IgG mAb administration 5 days after surgery. (* P<0.05 ANOVA, means±SD). B-D: n = 7 IgG MI, n = 5 anti-CD28 MI, n = 3 IgG sham, n = 4 anti-CD28 sham; * P<0.05 ANOVA, means±SD.
