# Supplementary material for: Treatment of mice with a ligand binding blocking anti-CD28 monoclonal antibody improves healing after myocardial infarction
Source: PLoS One. 2020 Apr 16;15(4):e0227734. doi: 10.1371/journal.pone.0227734 (PMC7161974; doi:10.1371/journal.pone.0227734)
Supplement: S1 Table — End-systolic area (ESA, mm2), end-diastolic area (EDA, mm2), end-systolic diameter (ESD, mm), end-diastolic diameter (EDD, mm), fractional area change (FAC, %). (PPTX) [file pone.0227734.s001.pptx]

## Slide 1
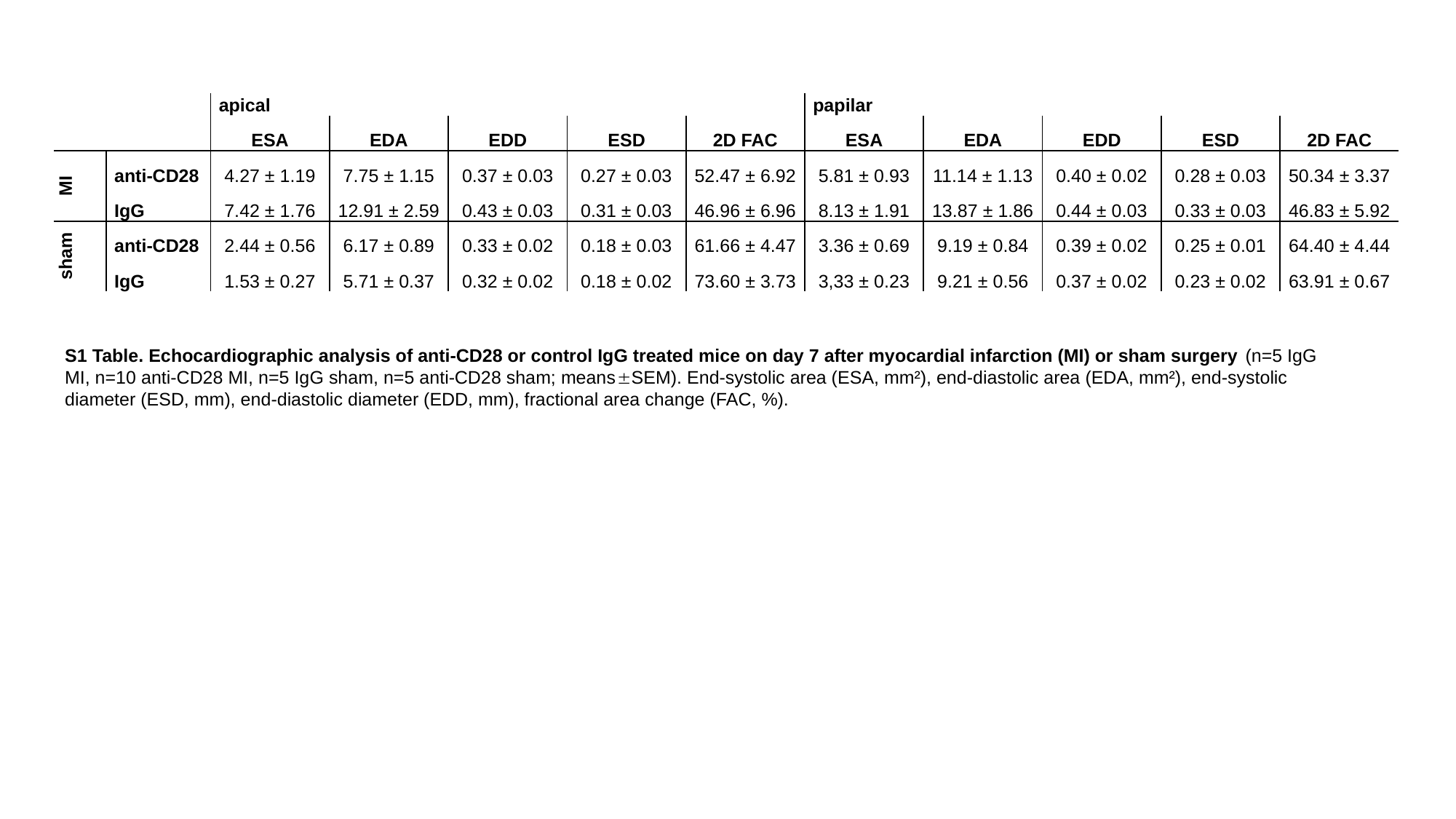

| | | apical | | | | | papilar | | | | |
| --- | --- | --- | --- | --- | --- | --- | --- | --- | --- | --- | --- |
| | | ESA | EDA | EDD | ESD | 2D FAC | ESA | EDA | EDD | ESD | 2D FAC |
| MI | anti-CD28 | 4.27 ± 1.19 | 7.75 ± 1.15 | 0.37 ± 0.03 | 0.27 ± 0.03 | 52.47 ± 6.92 | 5.81 ± 0.93 | 11.14 ± 1.13 | 0.40 ± 0.02 | 0.28 ± 0.03 | 50.34 ± 3.37 |
| | IgG | 7.42 ± 1.76 | 12.91 ± 2.59 | 0.43 ± 0.03 | 0.31 ± 0.03 | 46.96 ± 6.96 | 8.13 ± 1.91 | 13.87 ± 1.86 | 0.44 ± 0.03 | 0.33 ± 0.03 | 46.83 ± 5.92 |
| sham | anti-CD28 | 2.44 ± 0.56 | 6.17 ± 0.89 | 0.33 ± 0.02 | 0.18 ± 0.03 | 61.66 ± 4.47 | 3.36 ± 0.69 | 9.19 ± 0.84 | 0.39 ± 0.02 | 0.25 ± 0.01 | 64.40 ± 4.44 |
| | IgG | 1.53 ± 0.27 | 5.71 ± 0.37 | 0.32 ± 0.02 | 0.18 ± 0.02 | 73.60 ± 3.73 | 3,33 ± 0.23 | 9.21 ± 0.56 | 0.37 ± 0.02 | 0.23 ± 0.02 | 63.91 ± 0.67 |
S1 Table. Echocardiographic analysis of anti-CD28 or control IgG treated mice on day 7 after myocardial infarction (MI) or sham surgery (n=5 IgG MI, n=10 anti-CD28 MI, n=5 IgG sham, n=5 anti-CD28 sham; means±SEM). End-systolic area (ESA, mm²), end-diastolic area (EDA, mm²), end-systolic diameter (ESD, mm), end-diastolic diameter (EDD, mm), fractional area change (FAC, %).
