# Supplementary material for: Treatment of mice with a ligand binding blocking anti-CD28 monoclonal antibody improves healing after myocardial infarction
Source: PLoS One. 2020 Apr 16;15(4):e0227734. doi: 10.1371/journal.pone.0227734 (PMC7161974; doi:10.1371/journal.pone.0227734)
Supplement: S1 Fig — Representative plot and quantitative analysis of the four quadrants (B-E: n = 4 IgG MI, n = 5 anti-CD28 MI; all P> 0.05 between treatment groups, means±SD). (PPTX) [file pone.0227734.s002.pptx]

## Slide 1
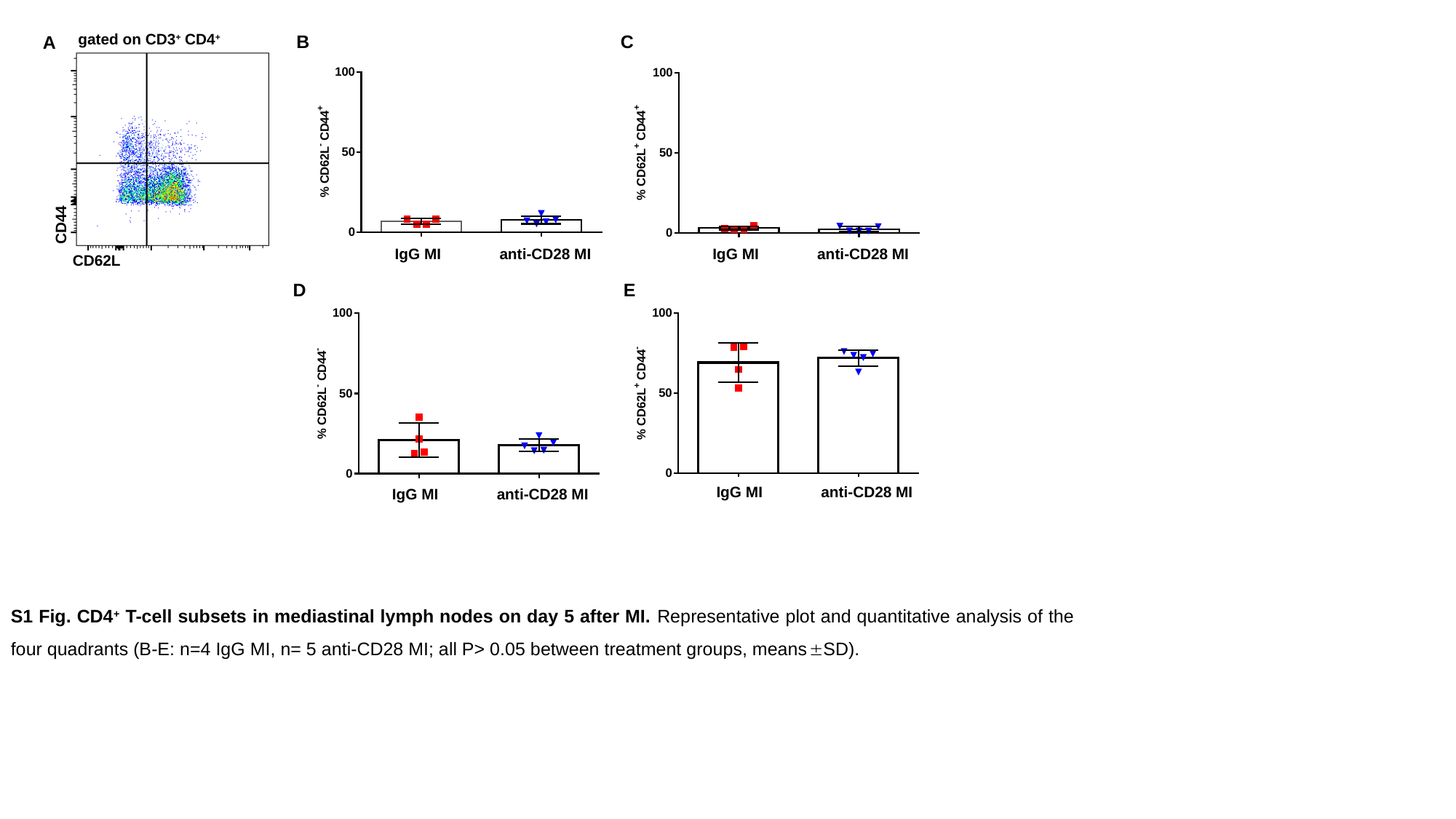

gated on CD3+ CD4+
CD44
CD62L
B
C
A
IgG MI anti-CD28 MI
D
E
IgG MI anti-CD28 MI
IgG MI anti-CD28 MI
IgG MI anti-CD28 MI
S1 Fig. CD4+ T-cell subsets in mediastinal lymph nodes on day 5 after MI. Representative plot and quantitative analysis of the four quadrants (B-E: n=4 IgG MI, n= 5 anti-CD28 MI; all P> 0.05 between treatment groups, means±SD).
