# Supplementary material for: Treatment of mice with a ligand binding blocking anti-CD28 monoclonal antibody improves healing after myocardial infarction
Source: PLoS One. 2020 Apr 16;15(4):e0227734. doi: 10.1371/journal.pone.0227734 (PMC7161974; doi:10.1371/journal.pone.0227734)
Supplement: S2 Fig — Representative Ladewig stainings of cardiac sections show collagen fibers in the infarct boarder zone in blue (red arrows in A). B: Myofibroblasts were immuno-stained with α-smooth muscle actin (brown). C: The semi-quantitative scoring of α-SMA showed no significant difference between anti-CD28 and IgG treated mice (n = 10 IgG MI, n = 11 anti-CD28 MI; n.s., t-test, means±SD). (PPTX) [file pone.0227734.s003.pptx]

## Slide 1
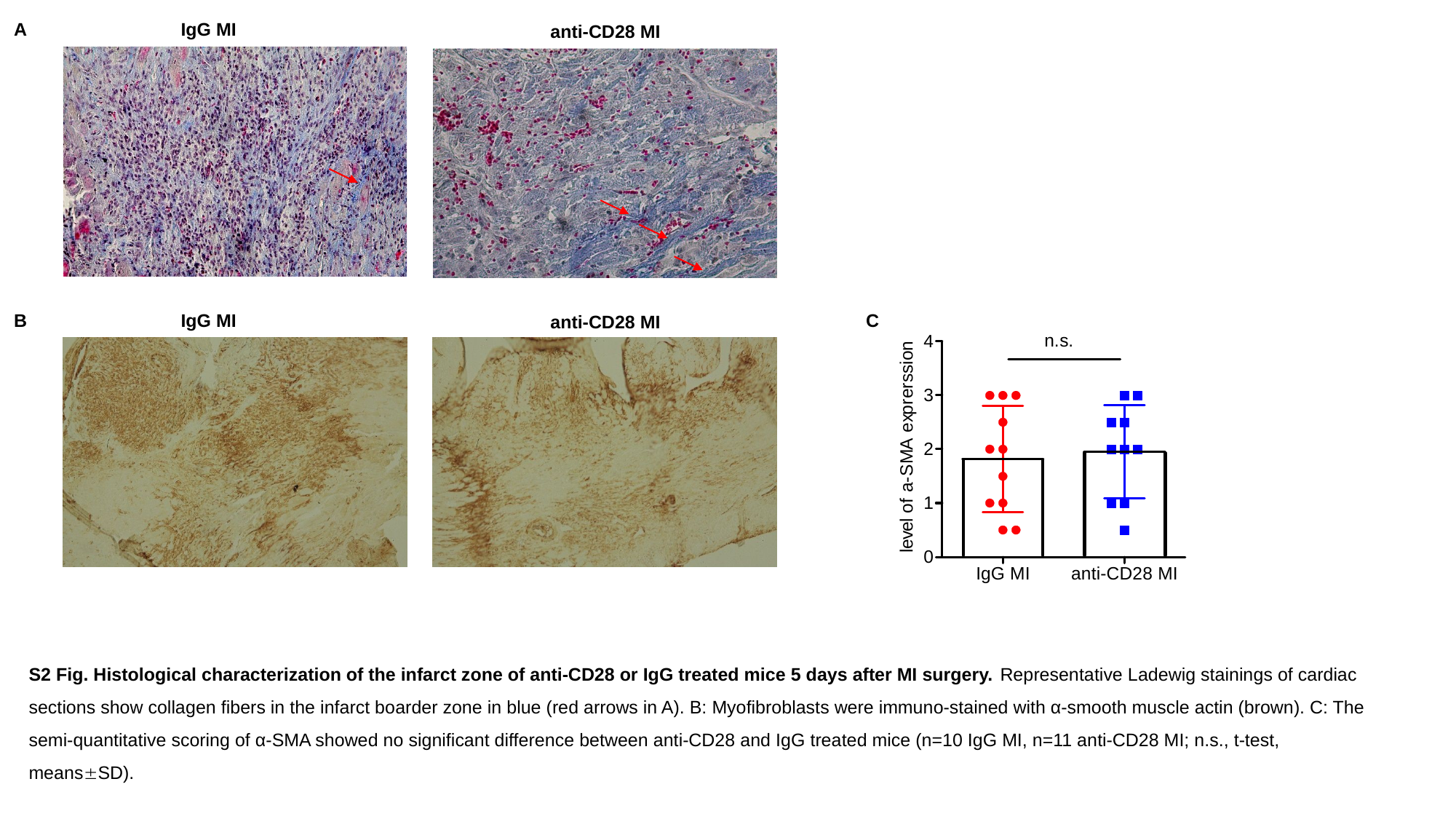

A
 IgG MI
 anti-CD28 MI
C
B
 IgG MI
 anti-CD28 MI
S2 Fig. Histological characterization of the infarct zone of anti-CD28 or IgG treated mice 5 days after MI surgery. Representative Ladewig stainings of cardiac sections show collagen fibers in the infarct boarder zone in blue (red arrows in A). B: Myofibroblasts were immuno-stained with α-smooth muscle actin (brown). C: The semi-quantitative scoring of α-SMA showed no significant difference between anti-CD28 and IgG treated mice (n=10 IgG MI, n=11 anti-CD28 MI; n.s., t-test, means±SD).
